# Beyond the CRAC: Diversification of ion signaling in B cells

**DOI:** 10.1111/imr.12770

**Published:** 2019-08-12

**Authors:** Trisha Mahtani, Bebhinn Treanor

**Affiliations:** ^1^ Department of Cell & Systems Biology University of Toronto Toronto Ontario Canada; ^2^ Department of Biological Sciences University of Toronto Scarborough Toronto Ontario Canada; ^3^ Department of Immunology University of Toronto Toronto Ontario Canada

**Keywords:** calcium, CRAC, ion channels, magnesium, TRP, zinc

## Abstract

Although calcium signaling and the important role of calcium release–activated calcium channels is well recognized in the context of immune cell signaling, there is a vast diversity of ion channels and transporters that regulate the entry of ions beyond calcium, including magnesium, zinc, potassium, sodium, and chloride. These ions play a critical role in numerous metabolic and cellular processes. The importance of ions in human health and disease is illustrated by the identification of primary immunodeficiencies in patients with mutations in genes encoding ion channels and transporters, as well as the immunological defects observed in individuals with nutritional ion deficiencies. Despite progress in identifying the important role of ions in immune cell development and activation, we are still in the early stages of exploring the diversity of ion channels and transporters and mechanistically understanding the role of these ions in immune cell biology. Here, we review the biology of ion signaling in B cells and the identification of critical ion channels and transporters in B‐cell development, activation, and differentiation into effector cells. Elucidating the role of ion channels and transporters in immune cell signaling is critical for expanding the repertoire of potential therapeutics for the treatment of immune disorders. Moreover, increased understanding of the role of ions in immune cell function will enhance our understanding of the potentially serious consequences of ion deficiencies in human health and disease.

## 1 INTRODUCTION

Ion channels act as critical gatekeepers, regulating the entry of ions such as sodium, calcium potassium, and magnesium into cells. While many are familiar with ion channels involved in the transport of calcium, and the important role of calcium as a second messenger in immune cell signaling, there are a multitude of ion channels, and together, they play a vital role in numerous metabolic and cellular processes. The importance of which is evidenced by the myriad of human disease pathologies associated with either ion deficiencies, or mutations in ion channel proteins, collectively referred to as channelopathies, and include both immunodeficiencies and autoimmune diseases. While the role of several ions and the interrogation of ion channels has been most extensively studied in the nervous system, we have also learned much about ion signaling and identified critical ion channels in T‐cell biology; however, much less is known about ion signaling in B cells. Thus, this review is focused on the key role ions and ion channels and transporters play in various stages of B‐cell life and function. Ions have been found to regulate B‐cell development, activation, and downstream effector functions in vivo. Here, we describe genetic and pharmacological evidence of these ions and ion channels in B cells and their importance in various aspects of B‐cell biology. This study is not meant to be a comprehensive review of the biophysical and structural properties of ion channels or their role in other cells of the immune system—where appropriate the reader is directed toward numerous excellent reviews on these topics.

## 2 DIVERSITY OF ION CHANNELS/TRANSPORTERS AND ION SIGNALING

Ions play a multitude of roles in cell development, maintenance, and activation. Anions, such as chloride (Cl^−^), and monovalent cations, such as sodium (Na^+^), potassium (K^+^), and hydrogen (H^+^), are known to regulate the membrane potential and their movement across the plasma membrane contributes to hyperpolarization and depolarization responses. Zinc (Zn^2+^), magnesium (Mg^2+^), and calcium (Ca^2+^) are stored in various cellular organelles and are released into the cytoplasm, as well as influxed from the extracellular space to maintain/replenish these stores and act as secondary messengers.

Ion channels and transporters are the predominant mechanisms of ion movement across the plasma membrane and the membranes of cell organelles. These proteins act as environmental sensors and can be activated by a variety of stimuli including stretch, osmotic pressure, temperature, and ligand or lipid binding.^1^, ^2^, ^3^, ^4^, ^5^ Ion channels move ions across membranes by passive transport over a concentration or electrical gradient, whereas ion transporters actively transport ions against these gradients. These proteins are expressed in a vast range of tissues and in both excitable and non‐excitable cells, indicative for their important role in regulating the membrane potential even in non‐excitable cells. There are many types of ion channels and transporters, which vary in the ions they transport, their selectivity, the stimuli that regulate them, and the role they play in physiological processes.^6^ Indeed, according to the HGNC database, there are over 320 ion channel genes in the human genome (http://www.genenames.org). These encode a diversity of ion channels and transporters categorized into a number of families including calcium‐activated potassium channels,^7^ voltage‐gated potassium channels,^8^ ryanodine receptors,^9^ voltage‐gated calcium channels,^10^ voltage‐gated sodium channels,^11^ GABA receptors,^12^ P2X ion channels,^13^ transient receptor potential (TRP) channels,^14^, ^15^, ^16^, ^17^ and volume‐regulated anion channels (VRAC).^18^, ^19^ There are also one‐member families including the voltage‐gated proton channel.^20^

**Figure 1:**
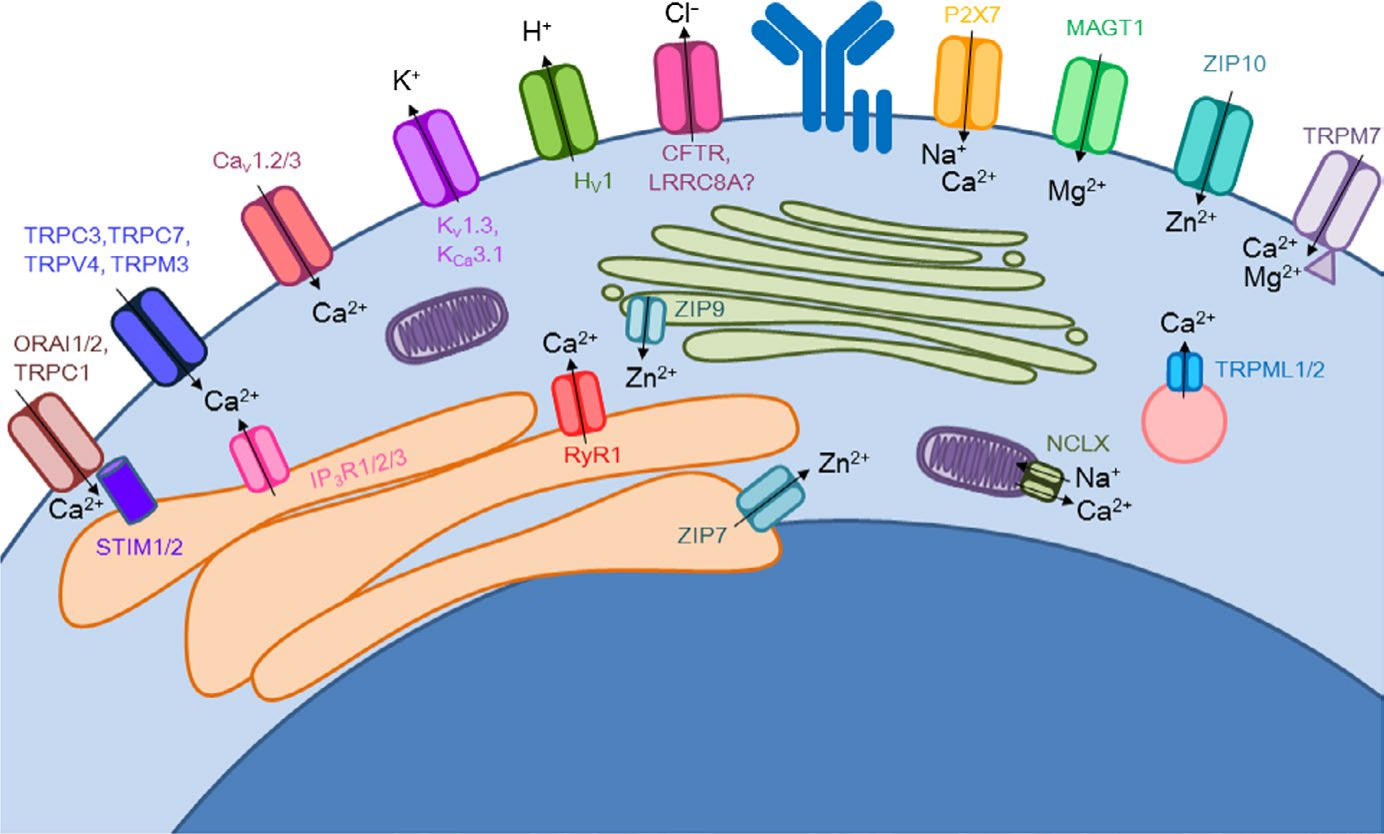
Functional ion channels expressed in B cells. Genetic and pharmacological evidence of ion channel activity in B cells provides evidence for their expression. B cells express store‐operated calcium entry channels on the ER (IP_3_R) and PM (CRAC, composed of ORAI and TRPC1, activated by STIM). Non‐selective cation channels from the TRP family, TRPC3, TRPC7, TRPM3, TRPV4, contribute to calcium influx upon various stimuli. Voltage‐gated calcium channels (Ca_v_1.2/3) have also been found to contribute to calcium influx in B cells. TRPML1/2 is found on lysosomes and regulates calcium concentrations by effluxing these ions into the cytosol. NCLX is a mitochondrial sodium‐calcium exchanger and contributes to cytosolic calcium levels from mitochondrial stores. Zinc stores are found in the Golgi and ER and zinc release into the cytosol is mediated by ZIP9 and ZIP7, respectively. Zinc is also influxed from extracellular media and ZIP10 on the plasma membrane contributes to this. TRPM7 and MAGT1 are both regulators of intracellular magnesium homeostasis and are found on the plasma membrane. TRPM7 has also been found to contribute to calcium store maintenance, as it is a non‐selective ion channel. P2X7, a purinergic receptor, is a non‐selective channel that has been found to influx sodium ions in B cells. Membrane potential changes upon B‐cell activation activate voltage‐gated potassium (K_v_1.3) and proton channels (H_v_1) to efflux these ions out of the cell. Additionally, anionic currents, namely chloride ions, have been identified in B cells potentially mediated by CFTR and LRRC8

Ion channels and transporters have been found to play a major role in regulating the development, maintenance, and activation of immune cells reviewed in 21, 22, 23, 24, 25, 26, 27, 28, 29, 30. However, we are still in the early stages of unraveling the diversity of ion channels and transporters expressed in immune cells during development, activation, and differentiation into effector cells and their crucial role in fine‐tuning immune responses. For instance, studies so far have only begun to explore about 10% of the potential 300+ ion channels and transporters in B cells (Figure 1). Nonetheless, what is emerging is that ion channels and transporters work in concert to regulate effective immune cell activation, and at the same time, limit hyperactivation and autoimmune responses. Elucidating the role of ion channels and transporters in immune cell signaling and identifying critical ion channels in the regulation of the immune response is critical for expanding the repertoire of potential therapeutics for the treatment of immune disorders, as ion channels are excellent pharmacological targets and there are already a number of ion channel and transporter specific inhibitors. In addition, increased understanding of the requirement for specific ions in immune cell function will enhance our understanding of the potentially serious consequences of ion deficiencies in human health and disease.

## 3 ION CHANNELS IN B‐CELL DEVELOPMENT

**Figure 2:**
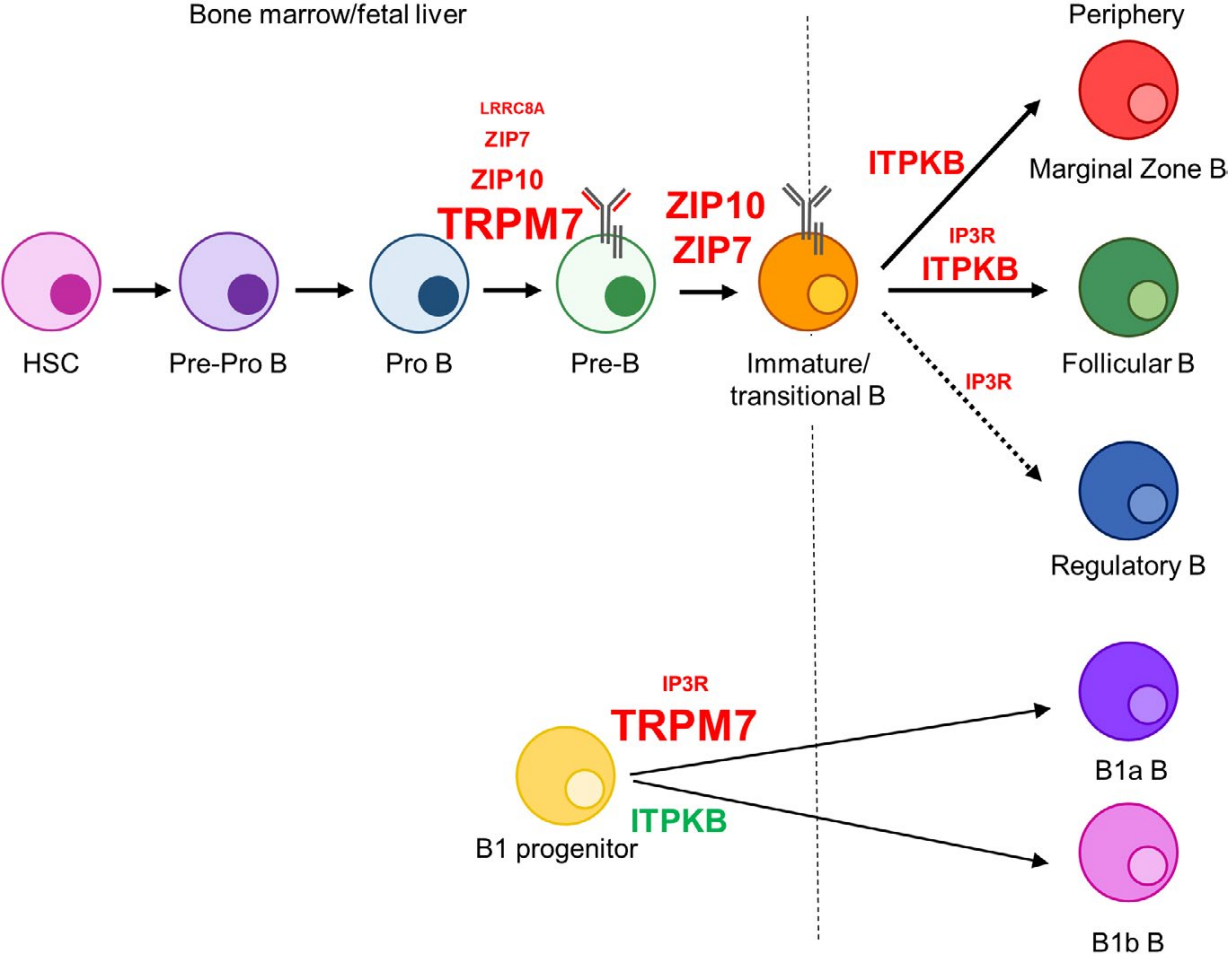
Ion channels in B‐cell development. Simplified schematic diagram of the stages of B‐cell development in the bone marrow and fetal liver and periphery (separated by dashed line), with key regulators highlighted in red that block B‐cell development or enhanced B‐cell development in green. The degree of impact on B‐cell development is indicated by the font size, with more severe impact on development in larger font and more modest impact on development represented by a smaller font

B‐cell development is orchestrated through a number of developmental stages characterized by processes including immunoglobulin (Ig) gene rearrangement, cell proliferation, and developmental checkpoints mediated by pre‐B‐cell receptor (pre‐BCR) and BCR signaling.^31^ B‐cell development begins in primary lymphoid tissue (eg, fetal liver and fetal/adult bone marrow) with the initial commitment of hematopoietic stem cells (HSCs) to pro‐B cells, in which heavy chain Ig gene rearrangement is initiated. Successful rearrangement of the heavy chain locus and pairing of mu heavy chain with the surrogate light chain to assemble the pre‐BCR is a critical checkpoint in B‐cell development, with successful pre‐BCR signaling necessary for developmental progression to pre‐B cells. During this phase, Ig light chain rearrangement commences and successful rearrangement and assembly of the BCR marks a second critical checkpoint in immature B cells. These immature B cells then leave the bone marrow to continue development through transitional stages, differentiating into mature follicular (FO) and marginal zone (MZ) B cells. In addition to these B‐cell populations, innate‐like B1 B cells, which develop in the fetal liver and primarily populate the peritoneal cavity, consist of B1a and B1b subsets, distinguished by the cell surface marker CD5 in the former. Collectively, these developmental processes require the concerted action of cytokines, transcription factors, and cell‐cell interactions, and consequently, the activation of numerous signaling pathways. Not surprisingly then, the regulation of intracellular ions plays an important, and in some cases essential, role in B‐cell development (Figure 2).

### 3.1 Zinc transporters

Some of the earliest work to highlight a role for ions and ion channels in B‐cell development came from studies examining nutritional deficiencies in human populations and the corresponding immune‐related phenotypes, many of which identified immune deficiencies, including lymphopenia in zinc deficient individuals.^32^, ^33^ To examine the role of zinc in immune cell development and function, rodent models of zinc deficiency were developed in which mice were fed a diet deficient in zinc to varying degrees.^34^ These studies demonstrated that zinc deficiency led to a significant loss in the proportion of B‐lineage cells in the bone marrow, with moderate zinc deficiency reducing B220^+^ cells by approximately 50% and severe zinc deficiency associated with nearly complete (>90%) loss of B220^+^ cells. Interestingly, early stages of B‐cell development, including pro‐ and pre‐B‐cell stages, were most severely affected by zinc deficiency, whereas immature B cells (B220^+^IgM^+^IgD^−^) and mature B cells (IgM^+^IgD^+^) were more resistant to zinc deficiency, albeit still grossly impacted by severe zinc deficiency.

Zinc is an essential trace element necessary for structural integrity of many proteins including enzymes and transcription factors.^35^ Indeed, zinc is required for the activity of some 300 enzymes and 2000 transcription factors. Thus, the regulation of zinc is crucial for numerous cellular processes. Zinc homeostasis is regulated by 14 *ZRT1- and IRT1-like protein* (ZIP) importers and 10 zinc transporter (ZnT) exporters that control the movement of Zn^2+^ between the cytosol and the extracellular space or cytoplasmic organelles reviewed in 36, 37. These transporters are differentially expressed in different immune cells,^38^ and most have yet to be investigated in the context of B‐cell development. Recently, however, two zinc transporters were identified as critical for B‐cell development.^39^, ^40^

ZIP10 is a plasma membrane ion channel that regulates the influx of Zn^2+^ from the extracellular space to the cytosol and was recently identified as important for the development of B cells.^40^ To investigate a role for ZIP10 in B‐cell development, Fukada and colleagues developed a murine model of B‐lineage specific deletion of ZIP10 under control of Mb1‐Cre,^41^ which mediates deletion from the pro‐B‐cell stage. These mice exhibit splenoatrophy and approximately 50% reduced numbers of mature peripheral CD19^+^ B cells, including transitional, MZ, and follicular B‐cell subsets. This reduction in mature peripheral B cells was attributed to a reduction in both pro‐B‐ and pre‐B‐cell populations in the bone marrow. To verify this phenotype was B cell intrinsic, the authors also utilized an inducible model of ZIP10 ablation by tamoxifen in cultured pro‐ and pre‐B cells and also found a reduction in pro‐ and pre‐B cells and increased apoptosis of these cells as measured by annexin‐V and induction of caspase‐3 activation. To investigate if ZIP10‐mediated uptake of zinc has an negative regulatory effect on caspase‐dependent apoptotic pathways, the authors examined the impact of intracellular zinc deprivation in the murine pro‐B cell line BAF‐B03, using the zinc selective chelator, *N*,*N*,*N*',*N*'‐tetrakis(2‐pyridylmethyl) ethylenediamine (TRPM), and found that this induced caspase activation and apoptosis, which could be reversed by zinc supplementation, indicating that zinc transported by ZIP10 is critical for suppression of caspase‐mediated apoptosis. In addition, the authors found that ZIP10 expression is altered by JAK‐STAT‐mediated cytokine signaling, suggesting a JAK‐STAT‐ZIP10‐zinc signaling axis in B‐cell development.

In a back‐to‐back accompanying paper, Fukada and colleagues deleted ZIP10 in mature B cells using Cre recombinase under control of the promoter of either the invariant chain (*Ii*; CD74), which is expressed in major histocompatibility class II (MHCII) expressing antigen‐presenting cells (APCs), including B cells, or CD21, which is expressed in mature B cells. Consistent with their accompanying data described above, deletion of ZIP10 in mature B cells resulted in a significant reduction in the number of FO B cells in the spleen and blood, and recirculating mature B cells in the bone marrow.^42^ In addition, the number of B1 B cells were also significantly reduced by deletion of ZIP10 using *Ii‐Cre*. Transfer of mature splenic B cells from ZIP10‐deficient mice into *Rag1*‐KO mice revealed a gradual loss of transferred B cells over time, indicating a B‐cell intrinsic defect, and suggesting that ZIP10 is required for persistence of mature B cells. Although the molecular mechanism for loss of mature B cells was not identified, enhanced BCR signaling in ZIP10‐deficient B cells (discussed below) was speculated to lead to B‐cell anergy and deletion.

More recently, Anzilotti et al^39^ identified compound heterozygous or homozygous rare variants in *SLC39A7* (ZIP7) in patients with early onset agammaglobulinemia and the absence of B cells. ZIP7 is a zinc transporter located in the endoplasmic reticulum (ER) that shuttles ER localized zinc into the cytoplasm.^38^ The examination of bone marrow of two patients revealed a progressive failure of B‐cell development with an excess of pro‐B cells relative to pre‐B cells. To further investigate the role of ZIP7 in B‐cell development, the authors utilized CRISPR‐Cas9 to introduce ZIP7 P198A mutation into C57BL/6 mice, orthologous to the most N‐terminal P190A mutation found in two independent kindreds. Mice homozygous for this mutation have drastically reduced late pre‐B cells, immature B cells, and recirculating mature B cells. Peripheral B‐cell numbers were also reduced in the spleen with progressive loss through transitional stages to FO and MZ B cells. T‐cell development and peripheral T‐cell numbers were normal, as were other leukocyte populations, indicating a B‐lineage specific requirement for ZIP7. Notably, supplementation of the drinking water with zinc could not rescue the developmental defect. The B‐cell intrinsic block in development was most pronounced from the late pre‐B to immature B‐cell stage, with a systematically altered pattern of gene transcription consistent with a developmental delay in pre‐B and immature B cells, but not at the earlier pro‐B‐cell stage; ZIP7‐deficient immature B cells continued to express *Rag* and *IL7r* genes and failed to upregulate genes associated with developmental progression such as *Tnfrsf13c* (BAFFR) and *Ms4a1* (CD20). These alterations in pre‐B and immature B cells were linked to alterations in pre‐BCR and BCR signaling (discussed below) and thus speculated to impact positive selection.

Collectively, these studies identified an important role for Zn^2+^ in B‐cell development and identified two essential zinc import channels. The identification of critical ion channels regulating zinc import in murine models has helped reconcile observations of immune deficiencies in human patients deficient in zinc. Although the molecular mechanism for the requirement for zinc has not been fully elucidated, one important mechanism appears to involve zinc‐mediated regulation of developmental checkpoints dependent on pre‐BCR and BCR signaling.

### 3.2 Transient receptor potential channels

The TRP family of ion channels is a large family of widely expressed ion channels that regulate the intracellular concentration of Ca^2+^, Mg^2+^, and trace metal ions, have diverse roles in cellular physiology, and respond to a variety of sensory stimuli such as taste, temperature, and pain reviewed in 15, 16, 17, 43. This superfamily of ion channels is classified into six subfamilies in vertebrates: TRPC (canonical), TRPV (vanilloid), TRPM (melastatin), TRPP (polysystin), TRPA (ankyrin), and TRPML (mucolipin), with different modes of activation and selectivity both within subfamilies and across the superfamily. Although many TRP channels are expressed in B cells,^44^ to date, only TRPM7 has been demonstrated as critical for B‐cell development.^45^

TRPM7 is divalent cation channel with selectivity for Ca^2+^, Mg^2+^, and Zn^2+^. Notably, TRPM7 and its closest relative, TRPM6, are the only known ion channels that contain an associated kinase domain, suggesting it may play an additional role in regulating cell signaling. The gene encoding TRPM7, *Ltrpc7*, was first cloned in an effort to identify novel Ca^2+^/cation channels expressed in hematopoietic cells.^46^ To further investigate the role of TRPM7 in cell physiology, the authors made use of the chicken B cell line, DT40; a cell line widely used in gene disruption studies due to its high rate of homologous recombination and rapid doubling time.^47^ Notably, targeted deletion of one allele was easily obtained; however, no clones were obtained with targeted disruption of both alleles, leading the authors to conclude that TRPM7 is required for cellular viability.^46^ To overcome this challenge, the authors took an inducible approach using a tamoxifen‐controlled Cre recombinase. The activation of Cre recombinase induced cell growth arrest and cell death within a 42‐ to 72‐hour time frame, again supporting the notion that TRPM7 is essential for cell survival. Subsequent studies demonstrated that the cell growth arrest and apoptosis of DT40 cells could be rescued by supplementation of the culture media with high concentrations of extracellular Mg^2+^, providing the first evidence that TRPM7‐mediated regulation of Mg^2+^ homeostasis may be important for cell survival.^48^

More recently, a murine model deficient in TRPM7 demonstrated that it is essential for embryonic development^49^; however, lineage‐specific deletion of TRPM7 does not necessarily lead to cell death in all cell types. For example, deletion of TRPM7 in T cells using Lck‐Cre resulted in a defect in thymocyte development, but nearly normal numbers of peripheral T cells.^49^ Moreover, Cre‐mediated deletion of TRPM7 in megakaryocytes increased the number of these cells in the bone marrow and red pulp of the spleen,^50^ and specific deletion in the myeloid lineage does not affect macrophage differentiation from bone marrow cells ex vivo.^51^

To examine a role for TRPM7 in B cells, we generated mice that lacked TRPM7 in the B‐cell lineage using TRPM7‐flox mice expressing Cre recombinase under the pan B cell *CD79a* (Mb1) gene promoter.^45^ We found a complete loss of B220^+^CD19^+^ mature B cells in the spleen, lymph nodes, and peripheral blood, as well as nearly complete loss of B1 B cells in the peritoneum in TRPM7‐deficient mice. Consistent with our findings of a lack of peripheral B cells, TRPM7‐deficient mice completely lacked immature, transitional, and mature B cells in the bone marrow. Indeed, we found a developmental block at the pre‐pro–B‐cell and pro‐B‐cell stages, coincident with promoter activity.^41^ The kinase function of TRPM7, however, was not required for B‐cell development, as knock‐in mice expressing a kinase dead mutant of TRPM7 had normal numbers of all B‐cell subsets. Notably, using an in vitro coculture system of HSCs that supports B‐cell development,^52^ we found a dose‐dependent increase in the number of B220^+^CD19^+^ cells derived from HSCs from TRPM7‐deficient mice supplemented with increased concentrations of extracellular Mg^2+^. However, even 10 mmol/L Mg^2+^, the concentration that supported development of TRPM7‐deficient DT40 B cells,^48^ development of B220^+ ^CD19^+^ cells from TRPM7‐deficient HSCs was still less than from wildtype (WT) HSCs. Our findings demonstrate that TRPM7 is essential for B‐cell development, and suggest that the Mg^2+^ transport function is most important. This is consistent with the observation that supplementation with high concentrations of extracellular Mg^2+^ is able to rescue the growth arrest seen in chicken DT40 B cells deficient in TRPM7.^48^ The discrepancy in the ability of supplementary Mg^2+^ to fully support primary murine B‐cell development^45^ compared to chicken DT40 B cells^48^ may be due to differential expression of other Mg^2+^ channels in DT40 vs primary HSCs; indeed, TRPM7‐deficient DT40 cells upregulate the Mg^2+^ transporter MagT1.^53^ Our findings also highlight, once again, distinct requirements for ion channels in different hematopoietic lineages. The requirement for TRPM7 may be more pronounced in B cells^45^ compared to T cells^49^ because T cells, but not B cells, express other Mg^2+^ permeable channels such as TRPM6.^44^ It may also be that the Mg^2+^ permeable channel, MagT1, has a more important role in T‐cell development.^54^

In light of the above discussion of the important role of zinc transporters in B‐cell development, and the permeability of TRPM7 to zinc ions, one might speculate that the B‐cell developmental defect we observe in TRPM7‐deficient mice might be due to a possible role for TRPM7 in Zn^2+^ transport. Although we did not test the ability of high concentrations of extracellular Zn^2+^ to support B‐cell development in our in vitro culture system of HSCs, the growth arrest observed in TRPM7‐deficient DT40 cells could only be reversed by supplementation with Mg^2+^, but not by Ca^2+^, Zn^2+^, Mn^2+^, or Ni^2+^,^48^ suggesting that lack of Zn^2+^ transport by TRPM7 is unlikely to account for our findings.

### 3.3 Volume‐regulated anion channel

Volume‐regulated anion channel is a heterooligomeric ion channel composed of leucine‐rich repeat (LRR)‐containing 8 (LRRC8) subunits (LRRC8A‐E) that transports chloride ions and various organic osmolytes, such as taurine and glutamate, across the plasma membrane reviewed in 18, 19. In 2003, Sawada et al isolated the *Lrrc8a* gene, which was found to be mutated in a patient with congenital agammaglobulinemia and a lack of B cells.^55^ To investigate the significance of the mutant LRRC8A, a vector encoding the mutant cDNA (resulting in a truncated protein) or control vector were used to infect bone marrow cells, which were then transferred into lethally irradiated mice to evaluate B‐cell reconstitution. Three months post transfer, mice infected with mutant bone marrow had reduced percentage of B and T cells, but not granulocytes or macrophages. Analysis of developing B‐cell subsets showed a developmental arrest at the pro‐B‐cell stage and a severe reduction in the proportion of pre‐B cells. Interestingly, in the patient, the unaffected *Lrrc8a* allele was transcribed and intact LRRC8A expressed; however, B cells were totally absent from peripheral blood, suggesting that the truncated form has a dominant‐negative effect. At the time of this finding, however, the role of LRRC8A in VRAC had not yet been identified so the molecular mechanism for loss of mature B cells was not investigated.

Subsequently, Kumar et al generated an *Lrrc8a* knockout mouse.^56^ LRRC8A is the most important subunit for channel function as suppression of LRRC8A nearly eliminated the presence of VRAC in mammalian cells.^57^, ^58^ In contrast to the patient with mutated *Lrrc8a*,^55^ LRRC8A‐deficient mice had modestly increased percentage of pro‐B cells, modestly decreased percentages of pre‐B cells, immature B cells , and recirculating mature B cells in the bone marrow.^56^ Splenic B220^±^ B‐cell numbers were reduced fourfold, although the proportions of FO B cells was relatively comparable to WT mice, whereas the proportions of transitional and MZ B cells was slightly decreased. There were no observed differences in peritoneal B1 B cells. Based on these observations, the authors concluded that LRRC8A plays a minor role in B‐cell development and peripheral B‐cell homeostasis. In contrast to the modest effect on B‐cell development, LRRC8A‐deficient mice exhibited a severe block in early thymic development, with a 10‐fold decrease in thymic cellularity, and an approximately threefold decrease in CD4^− ^CD8^−^ double‐negative (DN), CD4^+ ^CD8^+^ double‐positive, and CD4 and CD8 single‐positive thymocytes. The defect in T‐cell development was attributed to decreased proliferation and increased apoptosis of thymocytes. Curiously, the authors did not examine a role for VRAC channel function or a possible role for chloride ions; however in a subsequent follow‐up study examining a spontaneous mouse mutant designated ébouriffé (ebo), in which a 2‐bp deletion in *Lrrc8a* results in truncation of the 15 C‐terminal LRRs and dramatic reduction of VRAC activity, T‐cell development was normal.^59^ Thus, it seems that the role of LRRC8A in T‐cell development is independent of its channel function. The conflicting results observed in the patient with mutated *Lrrc8a* and *Lrrc8a^−/−^* mice with respect to B‐cell development may be due to the fact that the truncated LRRC8A found in the patient is transcribed with intronic sequences adjacent to the truncated seventh LRR, and perhaps these resulting additional 35 amino acids have a role in the loss of B cells, rather than VRAC activity, as suggested by Geha and colleagues.^59^

### 3.4 Calcium channels

Given the importance of calcium as a second messenger for many cell surface receptors, it seems likely that ion channels regulating Ca^2+^ efflux from the ER into the cytoplasm, or Ca^2+^ influx from the extracellular space into the cytosol would be important in B‐cell development. It was surprising then, that B‐cell development in the spleen, bone marrow, and peritoneal cavity was normal in mice deficient in the ER Ca^2+^ sensors, stromal interaction molecule 1 (STIM1) and STIM2, using Cre‐mediated deletion under control of *CD79a*.^60^ Similarly, deletion of the calcium release‐activated channel (CRAC) component ORAI1,^61^ or knock‐in of ORAI1 with a point mutation (R93W) that renders the pore‐forming subunit non‐functional,^62^ had no effect on B‐cell development. These findings are consistent with normal numbers of B cells in patients with mutations in *ORAI1* and *STIM1*.^63^, ^64^ Taken together, these findings demonstrate that B‐cell development does not require Ca^2+^ mediated through store‐operated calcium channels.

Recently, Tang and colleagues investigated the role of inositol 1,4,5‐trisphosphate receptor (IP_3_R)‐mediated Ca^2+^ release in B‐cell development.^65^ They generated a triple knockout of IP_3_R (IP_3_R1, IP_3_R2, and IP_3_R3 encoded by *Itpr1*, *Itpr2*, and *Itpr3*) designated IP_3_R‐TKO. They found total numbers of CD19^+^B220^+^ in the bone marrow were not significantly different in IP_3_R‐TKO mice compared to control mice. Consistent with this, numbers of pro‐, pre‐, and immature B cells were not altered. They did find, however, that the number of transitional B cells was increased and the number of recirculating mature B cells was reduced. In the periphery, the number of T2 cells was approximately double, whereas the number of FO B cells was reduced approximately 30%. There was no difference in the population of MZ B cells between IP_3_R‐TKO and control mice; however, the proportion of B1 B cells in the peritoneal cavity and the number of CD1d^high^CD5^+^ B10 B cells were both reduced. Thus, Ca^2+^ release mediated by IP_3_R in the ER plays an important role in some aspects of B‐cell development.

In addition to calcium permeable ion channels, Ca^2+^ homeostasis is also controlled by kinases that regulate the intracellular concentrations of inositol phosphates. Inositol‐1,4,5‐trisphosphate 3‐kinase B (Itpkb) regulates the concentration of key inositol phosphates involved in Ca^2+^ homeostasis by phosphorylating inositol‐1,4,5‐trisphosphate (IP_3_), converting it to inositol‐1,3,4,5‐tetrakisphosphoate (IP_4_). To investigate the function of Itpkb in B‐cell development and function, Miller and colleagues^66^ utilized an *Itpkb*‐deficient mouse model generated by the random germline mutagen *N*‐ethyl‐*N*‐nitrosourea.^67^ Deletion of Itpkb had no effect on B‐cell subsets in the bone marrow, except for recirculating mature B cells, which were reduced by approximately 50%. Consistent with this observation, splenic B‐cell numbers were reduced 80%, with decreases in transitional, FO, and MZ subsets. In contrast, B1 B cells were increased approximately 50%. The authors also found Itpkb‐deficient B cells had altered BCR signaling (discussed below) and therefore proposed that Itpkb and its product, IP_4_, are negative regulators of BCR signaling and deficiency in Itpkb leads to enhanced negative selection.

Taken together, the findings discussed here implicate Zn^2+^ and Mg^2+^ as necessary ions for B‐cell development, and suggest that Ca^2+^ has less of a role in B‐cell development, albeit still being important for positive and negative selection of B cells through regulation of BCR and pre‐BCR signaling.

## 4 ION SIGNALING UPON BCR STIMULATION

### 4.1 Store‐operated calcium entry

**Figure 3:**
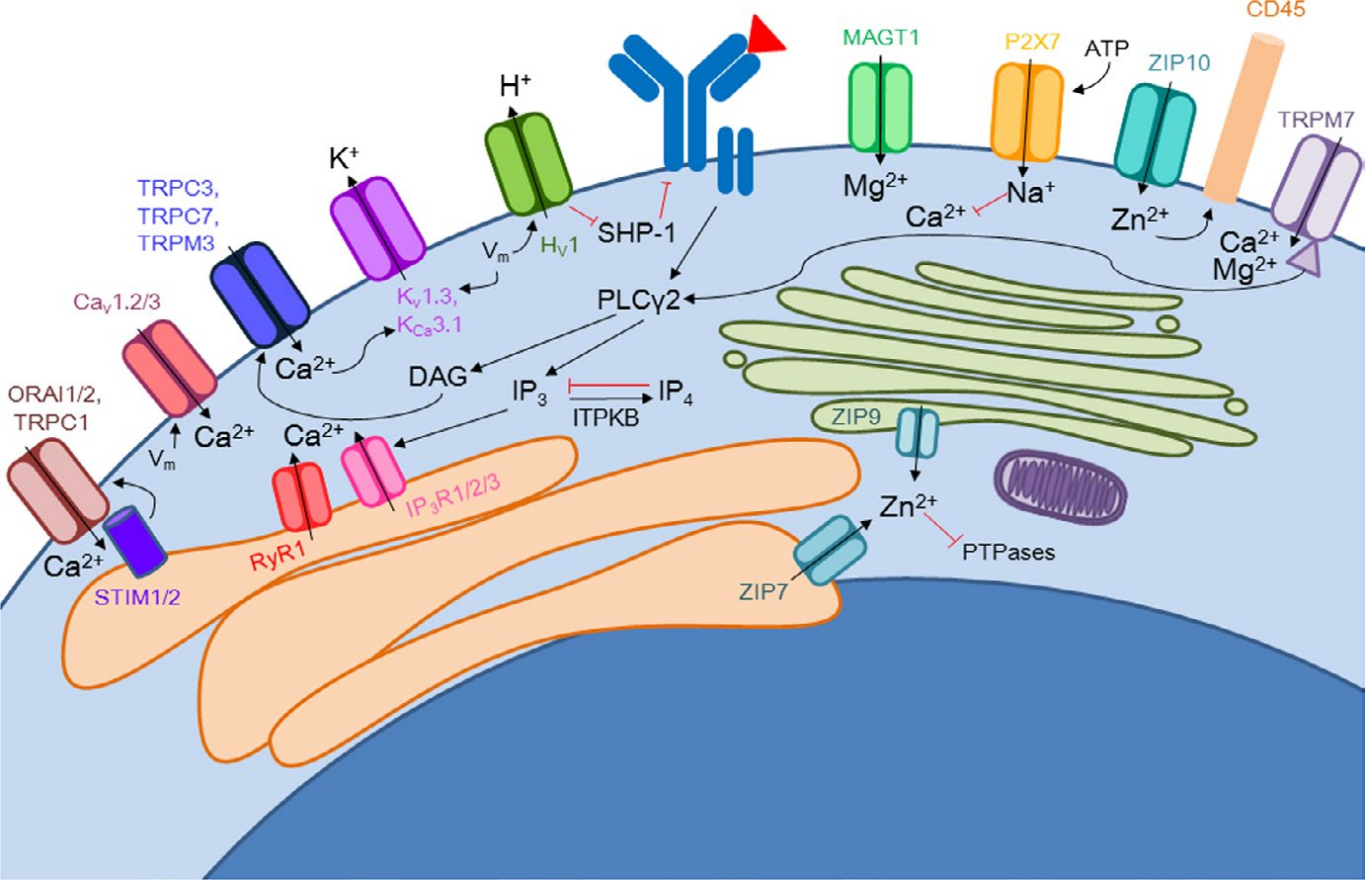
Ion channel activation and function during B‐cell activation. B cells recognize antigen with their B‐cell receptor (BCR) and signaling cascades are initiated that are fine‐tuned for optimal B‐cell activation. Genetic and pharmacological studies have demonstrated that the kinase domain of transient receptor potential melastatin 7 (TRPM7) phosphorylates phopholipase C gamma 2 (PLCγ2) and once activated, it can hydrolyze phosphatidylinositol 4,5‐bisphosphate (PIP_2_) to produce inositol 1,4,5‐trisphosphate (IP_3_) and diacylglycerol (DAG). IP_3_ binds to IP_3_ receptors (IP_3_Rs) on the endoplasmic reticulum (ER) and stimulates the release of calcium stores by IP_3_R and ryanodine receptors (RyR). IP_3_ conversion to inositol 1,3,4,5‐tetrakisphosphate (IP_4_) by IP_3_ kinase B (ITPKB) regulates the degree of calcium signal upon activation. Stromal interaction molecules (STIM) recognize a depletion of ER stores and activate calcium release activated calcium (CRAC) channels comprised of ORAI subunits and TRPC1. DAG also activates store‐independent calcium influx channels TRPC3 and TRPC7. Additionally, TRPM3 and Ca_v_ channels are activated and contribute to calcium influx. P2X7 receptors are activated by extracellular ATP and dampen calcium signals upon B‐cell activation by influx of sodium ions. Zinc release from Golgi (ZIP9) and ER (ZIP7) stores into the cytoplasm inhibits phosphatases and dampens signaling, whereas ZIP10 positively regulate CD45 phosphatase activity. Proton (Hv1) and potassium efflux (K_Ca_3.1, K_v_1.3) upon membrane hyperpolarization are both regulated by the membrane potential changes or calcium entry into the cell

Stimulation of a B cell through engagement of the BCR induces receptor clustering and initiation of signaling pathways. These pathways include the release of ion stores, changes in membrane potential, movement of ions across the membrane, and recovery of emptied ion stores. These ions act as secondary messengers and are important for effective BCR signaling (Figure 3). The most classically profiled secondary messenger is calcium, as the activation of phospholipase C gamma 2 (PLCγ2) leads to hydrolysis of phosphatidylinositol 4,5‐bisphosphate (PIP_2_), a membrane localized lipid, and production of IP_3_ and diacylglycerol (DAG).^68^ IP_3_ binds to IP_3_ receptors on the ER and causes the release of calcium stores from the ER.^68^ Once these stores have emptied, STIM1 and STIM2 are activated and can, in turn, activate CRAC channels composed of ORAI1 and ORAI2 subunits on the plasma membrane to replenish calcium stores.^68^ This process, known as store‐operated calcium entry (SOCE), is thought to govern the calcium response in B cells.

Indeed, knocking out all three IP_3_ receptors (IP_3_R‐TKO) in murine B cells leads to essentially no calcium flux upon BCR stimulation.^65^ Both phases of calcium signaling, the initial peak from the emptying of ER stores, and the sustained flux in the presence of extracellular calcium were diminished in IP_3_R‐TKO B cells. However, treating cells with thapsigargin, a drug that empties calcium stores by inhibiting the sarco/ER calcium ATPase (SERCA), showed no difference between IP_3_R‐TKO and WT B cells. This suggests that IP_3_Rs are crucial for calcium signaling in response to BCR stimulation, but are dispensable for maintenance of calcium stores.

Similarly, a murine double knockout (DKO) of STIM1 and STIM2 also led to significantly reduced calcium flux upon BCR stimulation in both the peak and sustained phases.^60^ Single gene knockouts demonstrated that STIM1 is more important than STIM2 for both maintenance of stores and calcium influx into cells, as STIM2‐KO B cells do not show much of a defect in comparison to the DKO, whereas STIM1‐KO B cells do.^60^ The abrogation of calcium flux seen in the DKO signifies the key role of these proteins in regulating SOCE currents. Previous findings in DT40 B cells are in agreement, as overexpressing STIM1 markedly enhances calcium influx upon BCR stimulation.^69^ However, in the murine STIM1/2‐DKO,^60^ unlike the IP_3_R‐TKO,^65^ treatment of cells with thapsigargin revealed a defect in ER store filling in DKO B cells, as Ca^2^^+^ flux was greatly reduced. Therefore, STIM1/2 are critical for the maintenance of Ca^2+^ stores and their absence results in reduced ER calcium release, as well as reduced calcium uptake upon BCR stimulation.

It was also found that CRAC channels behave similarly in chicken DT40 B cells, as knocking out ORAI1 had no effect on SOCE currents, whereas knocking out ORAI2 actually increased calcium entry.^70^ Knocking out both of the ORAI subunits in DT40 cells completely abrogated the second phase of SOCE currents.^70^ This suggests that CRAC channels may be composed of ORAI subunits together with other channel proteins to compensate for the lack of one subunit; however, loss of both subunits prevents the formation of CRAC channels.

As discussed above, IP_3_ generated during the SOCE response can also be converted to IP_4_ by ITPKB‐mediated phosphorylation, and this conversion has been found to dampen SOCE in mast cells.^71^ Deletion of *Itpkb* in a murine model enhanced calcium signaling upon BCR stimulation, especially in the presence of extracellular calcium.^66^ This was specific for BCR stimulation, as there was no difference in Ca^2+^ store release upon thapsigargin treatment. Therefore, ITPKB or its product, IP_4_, regulates SOCE channels to control the amount of calcium that enters B cells upon BCR stimulation.

In addition to the conventionally studied players in SOCE, TRP channels may also play a role in the composition of heterogeneous CRAC channels and maintenance of calcium stores. For example, deletion of TRPC1 in DT40 B cells impacted both the peak and sustained Ca^2+^ flux upon BCR stimulation.^72^ Treatment of cells with thapsigargin demonstrated that TRPC1 does not play a role in ER Ca^2+^ release, but is involved in extracellular Ca^2+^ entry upon ER store emptying. This finding demonstrated that, at least in B cells, other subunits may be incorporated as components of SOCE channels. In addition, TRPM7 has also been shown to be important for Ca^2+^ flux upon BCR stimulation, as SOCE currents in the presence of extracellular calcium are reduced in TRPM7‐KO DT40 B cells.^70^ Interestingly, the kinase domain of TRPM7 was found to be important for refilling and maintaining calcium stores, as both store content at steady‐state and rate of filling after release were impacted in DT40 B cells expressing a kinase dead mutant of TRPM7.^70^ This finding may be related to the role of TRPM7 kinase in phosphorylation of PLCγ2,^73^ although how this impacts PLCγ2 activity is not currently clear.

### 4.2 Non‐store‐operated calcium channels

Transient receptor potential channels have also been found to play a role in store‐independent calcium responses. These responses are characterized by the activation of plasma membrane calcium channels through direct interactions with secondary messengers or byproducts of PLCγ2 activation. For example, TRPC3 has been found to be important for DAG‐mediated calcium responses upon BCR stimulation.^74^, ^75^, ^76^ This was demonstrated both by the loss of DAG analog, 1‐oleoyl‐2‐acetyl‐sn‐glycerol (OAG)‐induced currents in TRPC3‐mutant cells, as well as a reduction in Ca^2+ ^flux upon BCR stimulation.^74^ Consistent with this, the overexpression of TRPC3 in DT40 B cells increases the second phase of Ca^2+^ flux upon BCR stimulation and induces a current in the presence of OAG that is not present without TRPC3 expression.^75^, ^76^ OAG treatment can also induce TRPC3 currents in IP_3_R knockout DT40 B cells, suggesting a SOCE mechanism does not activate it.^75^, ^76^ However, it may be that TRPC3 is also important for SOCE currents, as Ca^2+^ flux upon thapsigargin treatment was higher in both WT and IP_3_R‐KO DT40 B cells expressing human TRPC3.^75^ This study also showed TRPC3‐dependent OAG‐induced currents, and these were actually higher in IP_3_R‐KO DT40 cells, suggesting the lack of IP_3_R allows for enhanced activation of TRPC3 and a potential dual role of this channel in B cells.^75^ In addition to the role of TRPC3 in non‐SOCE, deletion of TRPC7 in DT40 B cells demonstrated a lack of a clear, detectable non‐store‐operated calcium current in the presence of gadmium, a SOCE current inhibitor, upon BCR stimulation, indicating a role for this channel in this pathway as well.^77^ Interestingly, the lack of TRPC7 also affected calcium stores, as they were enlarged in the absence of the channel. It has also been seen that chronic fatigue syndrome patients have reduced expression of TRPM3 on the surface of their B cells, and this leads to a significantly decreased calcium flux in response to treatment with thapsigargin and crosslinking of the BCR and CD21.^78^

Interestingly, B cells have also been found to express calcium channels typically found on excitable cells, such as neurons or muscle cells. Their presence on non‐excitable cells suggests they have multiple gating properties, as they have been found to contribute to calcium responses despite the lack of evidence of voltage‐gated calcium channel currents by electrophysiology. For example, primary human CD19^+^, DAKIKI (human IgA^+^ B cell line) were found to express ryanodine receptor 1 (RyR1) and DT40 B cells were found to express both RyR1 and RyR3.^79^, ^80^ Ryanodine receptors, similar to IP_3_Rs, mediate release of calcium ions from the ER, but are primarily found in skeletal and cardiac muscle cells and neurons.^9^ Stimulation of RyR with 4‐chloro‐*m*‐cresol (4‐CMC) in primary human CD19^+^ and DAKIKI B cells induced calcium currents and these currents were amplified in the presence of ryanodine (a poisonous diterpenoid found in the South American plant *Ryania speciosa* that has high affinity for RyR and for which the channel was originally named) at concentrations (nmol/L) that promote the receptor to stay in a half‐open state.^80^ BCR stimulation in calcium‐free media with egtazic acid/ethylene glycol‐bis(β‐aminoethyl ether)‐*N*,*N*,*N*′,*N*′‐tetraacetic acid) (EGTA) and ryanodine exhibited much heightened calcium flux compared to cells stimulated in the absence of ryanodine.^79^ The use of RyR inhibitors, ruthenium red (RuR) and ryanodine at μmol/L concentrations, which completely shut the channel, on DT40 B cells reduced SOCE in response to ionomycin with and without extracellular calcium.^79^ Additionally, RyR activators such as cyclic ADP ribose (cADPR) led to more rapid calcium currents compared to control EGTA treatment, whereas RyR inhibitors such as RuR and 8‐*N*‐cADPR led to decreased and slower calcium currents compared to control treatment.^79^ Therefore, RyR receptors play a significant role in the release of calcium stores from the ER in B cells upon stimulation.

In addition to RyR receptors, a non‐voltage‐gated L‐type calcium channel is expressed in primary rat B cells and human B lymphoma cell lines.^81^, ^82^ L‐type calcium channels are a family of voltage‐dependent calcium channels (the “L” standing for the long‐lasting currents observed for these channels) composed of Ca_v_1.1, Ca_v_1.2, Ca_v_1.3, and Ca_v_1.4 subunits. Again, these channels are typically associated with excitable cells; however, a truncated form of L‐type channels has been found in non‐excitable cells, and is generally thought to be voltage‐insensitive reviewed in 83. In primary rat B cells, treatment with specific L‐type calcium channel inhibitors, nicarpidine and calciseptine, completely abrogated the expected calcium peak response upon BCR stimulation, which was found to be Ca_v_1.3 dependent.^82^ Calcium responses upon BCR stimulation were also significantly reduced in human B cell lines upon treatment with the inhibitors of L‐type calcium channels, diltiazem, verapamil, and nifedipine, and in at least one cell line, truncated Ca_v_1.2 was found to be the L‐type channel expressed.^81^ Treatment of rat B cells with an L‐type calcium channel agonist, Bay K 8644, induced a much longer peak calcium response,^82^ whereas in human B‐cell lymphoma lines, adding this agonist prior to BCR stimulation had no effect, but treatment post‐BCR stimulation induced an abrupt change from peak to sustained phases of calcium signaling.^81^ It was also found that the current induced by this L‐type calcium channel is a result of increased cyclic guanosine monophosphate (cGMP) levels after BCR stimulation and that inhibiting guanylyl cyclase, which synthesizes cGMP from GTP, with LY83583 led to a greatly reduced BCR‐induced calcium response.^82^ Additionally, the majority of the L‐type calcium channels (Ca_v_1.2‐4) are expressed in DT40 B cells, and inhibiting these channels demonstrated the same disruption in calcium signaling upon BCR stimulation.^83^ However, unlike in T cells, Ca_v_ channels in DT40 B cells are only important for the sustained phase of calcium signaling and not for thapsigargin‐sensitive stores.^83^ Interestingly, deletion of Ca_v_1.3 in DT40 B cells was found to be lethal; however, there was no impairment in calcium signaling upon BCR stimulation using tamoxifen‐inducible deletion of Ca_v_1.3.^83^ This suggests a role for other L‐type channels in calcium signaling in this process and that L‐type calcium channels are activated by differential stimuli in lymphocytes and are important to establishing calcium currents upon antigen receptor activation.

### 4.3 Unconventional ions

As outlined above, two other divalent cations, magnesium and zinc, both play very important roles in the development and maintenance of B cells. However, not much is known about the role these ions play during BCR stimulation. It has been shown that Mg^2+^ does appear to increase in cytoplasmic concentration after BCR stimulation and is dependent on calcium influx; however, the kinetics are gradual and monophasic with no clear peak or drop‐off.^84^ Deletion of the Mg^2+^ permeable channel, TRPM7, in DT40 B cells lowers intracellular Mg^2+^ levels at resting state, and is associated with impaired growth of these cells, as high concentrations of extracellular Mg^2+^ can rescue this defect.^48^ Similarly, murine B cells deficient in the Mg^2+^ channel, MAGT1, had reduced magnesium levels in both resting cell and upon activation.^85^ Surprisingly, these cells also had increased calcium influx in both phases of SOCE upon BCR stimulation; however, there were no differences in calcium flux upon thapsigargin treatment, suggesting that regulation of Mg^2+^ plays a key role during BCR signaling. As magnesium is important for the function of many enzymes, it is perhaps not surprising that store release and extracellular influx upon BCR stimulation would be crucial to downstream events and differentiation; however, further investigation of the role of Mg^2+^ in BCR signaling is required to elucidate the specific requirements and downstream targets of Mg^2+^.

It has also been shown that cytoplasmic Zn^2+^ levels increase over a 10‐minute period upon BCR stimulation with no drop‐off,^86^ and activated CD69^+^ B cells have increased Zn^2+^ levels.^87^ Knocking out ZIP9, a zinc transporter found in the Golgi, in DT40 B cells impaired the intracellular rise in Zn^2+^ upon BCR stimulation.^86^ Similarly, ZIP10 has also been shown to be important for Zn^2+^ influx in developing B cells and activation of mature naive B cells suggesting a crucial role for Zn^2+^ flux upon BCR stimulation.^42^ While these studies importantly identify transporters mediating Zn^2+^ flux in B cells, further studies are required to understand the role of zinc in B‐cell activation and its impact on downstream events.

### 4.4 Monovalent ions

B cells are non‐excitable cells, which do not exhibit activation upon membrane depolarizing conditions. However, upon BCR stimulation, they do undergo differential membrane potential and require tight regulation of membrane polarization.^88^, ^89^, ^90^ Monovalent cation flux in B cells upon BCR stimulation is not well characterized; however, Na^+^, K^+^, and H^+^ currents have been detected during BCR stimulation. For example, voltage‐gated hydrogen channel 1 (*HVCN1*)‐deficient murine B cells had no detectable H^+^ currents at steady state regardless of extracellular conditions and exhibited an indirect effect of impaired H^+^ homeostasis upon BCR stimulation, as local ROS production at the membrane was decreased.^91^

Potassium currents have also been observed in B cells^92^ and are attributed to K_v_1.3, K_Ca_3.1, and TREK‐2 channels, which are expressed in B cells.^90^, ^93^, ^94^, ^95^, ^96^ Such a potassium current can be induced through K_Ca_3.1 by treatment of B cells with ionomycin^90^ or BCR stimulation,^88^ as this current is dependent on the influx of calcium.^90^ Inversely, blocking potassium efflux, or supplementing extracellular media with potassium^88^ was found to reduce calcium influx upon BCR stimulation.^90^ These findings suggest a cyclical regulation of these two ions, whereby Ca^2+^ influx activates K^+^ channels dependent on this ion for their activation, thereby hyperpolarizing the cell and increasing the driving force for more Ca^2+^ entry. Consistent with this idea, B cells transiently hyperpolarize upon BCR stimulation^88^; however, agonist induced K_Ca_3.1 current upon BCR stimulation led to prolonged hyperpolarization but unexpectedly, the authors did not see an increase in calcium currents.^97^ Thus, further studies are needed to understand the codependence of Ca^2+^ and K^+^ currents on each other upon activation, as the activating cue for non‐calcium‐dependent potassium channels is not well understood in B cells.

Sodium currents have not been well characterized in B cells; however, a few studies suggest that Na^+^ flux may also play a role in B‐cell responses. The first evidence suggesting a role for Na^+^ channels in B cells came from patch‐clamp experiments that demonstrated Na^+^ conductance in B cells upon stimulation in calcium‐free media,^88^ and the subsequent immunoprecipitation of a protein complex using an antibody specific for a bovine kidney Na^+^ channel.^98^ Additionally, P2X7, a purinergic receptor, has been found on B cells and was shown to regulate Na^+^ influx upon BCR stimulation, and dampen calcium influx as a result.^89^ Similar findings have been shown in T cells with TRPM4, as Na^+^ influx depolarizes the cells upon activation and regulates calcium influx.^99^ This depolarization from Na^+^ influx is thought to dampen calcium currents and activate voltage‐gated potassium channels, which would return the membrane to a hyperpolarized state and allow for more calcium influx.^99^ This “see‐saw” effect between depolarized and hyperpolarized membrane potential is what leads to the observed calcium oscillations. This suggests a crucial role for Na^+^ in regulating B‐cell activation and preventing enhanced calcium responses which can direct the cell toward apoptosis rather than proliferation and differentiation.

Anionic currents in B cells are also not well understood and B cells express very little of the ATP‐gated anion channel, CFTR. Indeed, antisense oligonucleotide blocking of gene transcription has been the only way to determine the presence and chloride current activity of the CFTR channel in B cells.^100^ It has, however, been found that at resting state, B cells do express a large conductance anionic channel with preference for chloride ions, and electrophysiology demonstrated the channel preferentially transports Cl^−^ over both K^+^ and Na^+^ when these ion were present on the intracellular side of the membrane.^101^ However, when chloride was replaced by aspartate, channel reversal potential and conductance were not altered suggesting that this channel is permeable to larger anions. Interestingly, B cells have been found to not undergo regulatory volume decrease in hypotonic conditions, which is characteristic of cells that swell and reduce swelling by effluxing potassium and chloride ions.^102^ This was found to be due to the lack of potassium efflux upon swelling^102^; however, B cells did exhibit a chloride current comparable to other cells.^103^ Additionally, this channel conductance can be blocked by inhibitors, stilbene derivatives disodium 4‐acetamido‐4′‐isothiocyanato‐stilben‐2,2′‐disulfonate (SITS) and 4,4'‐diisothiocyano‐2,2'‐stilbenedisulfonic acid (DIDS), of chloride flux and the channel responsible was found to be voltage sensitive.^104^ This suggests that chloride currents may play a role in regulating membrane potential after BCR stimulation, as other ion fluxes polarize the cell membrane.

## 5 ION CHANNELS IN BCR ACTIVATION AND SIGNALING

Ion channels play a key role in fluxing secondary messengers and cofactors for protein activity and defects in ion concentrations during both steady‐state and activating conditions can lead to abnormal signaling downstream of the mitogenic receptor. Ion dysregulation has been found to have impacts on signaling downstream of the BCR, which can lead to defects in processes that lead to humoral immunity. In the following sections, we discuss the evidence of ions and ion channels that play a key role in BCR signaling and B‐cell activation (Figure 3).

### 5.1 Store‐operated and non‐store‐operated calcium channels

Defects in SOCE currents in B cells lead to downstream signaling defects differentially depending on the targeted protein. In IP_3_R‐TKO murine B cells, NFAT dephosphorylation is completely abrogated, but ERK and JNK signaling are increased compared to control B cells.^65^ These cells also have impaired proliferation and survival when stained with carboxyfluorescein succinimidyl ester and propidium iodide (PI) after BCR stimulation. Thus, impaired signaling upon BCR stimulation as a result of diminished calcium leads to impaired B‐cell activation. STIM1/2 DKO B cells also have impaired NFAT dephosphorylation, but surprisingly do not show any differences in phosphorylation of other proteins involved in BCR signaling.^60^ Similar to IP_3_R‐TKO B cells, STIM1/2‐DKO B cells do, however, fail to survive and proliferate after BCR stimulation. These studies demonstrate that SOCE is crucial to responses to BCR stimulation, as there is a marked reduction in surviving B‐cells poststimulation. Consistent with these studies, knocking out TRPC1, which also plays a role in the SOCE currents, in DT40 B cells also led to decreased NFAT activity.^72^

In contrast, ITPKB‐KO B cells have increased calcium signaling and slightly reduced PLCγ2 phosphorylation despite normal levels of IP_3_ generation.^66^ These cells did not upregulate costimulatory molecules and activation markers CD86, MHC class II, and CD69, and failed to proliferate compared to control cells upon BCR stimulation. They were also blocked from progressing to the G1 stage of the cell cycle post‐BCR stimulation. This is in agreement with the finding that overexpressing STIM1 in DT40 B cells led to enhanced calcium signaling upon BCR ligation, which led to enhanced ERK phosphorylation and increased cell death.^69^ This highlights the important fine‐tuning of SOCE responses upon B‐cell activation, as enhanced SOCE responses can lead to signaling defects and induction of apoptotic pathways.

Defective calcium influx from non‐store‐operated pathways can also impact B‐cell activation. For example, expressing a truncated form of TRPC3 defective in channel function in DT40 B cells led to defective PLCγ2 and PKC translocation to the plasma membrane upon BCR stimulation, and ERK phosphorylation and NFAT activity were also reduced.^74^ Similarly, BCR stimulation in the presence of ATP‐activated P2X7 receptors and were found to influx Na^+^ and dampen Ca^2+^ currents, which led to defective NFAT activation.^89^ Indeed, calcium ionophore treatment of B cells in the absence of any other stimulation elicits MAP kinase activation solely through Ca^2+^ influx, indicating the key role for calcium signaling in distal B‐cell signaling.^105^ Similarly, human B cells require extracellular calcium for multiple signaling proteins involved in B‐cell activation, as exposure to extracellular calcium without BCR stimulation increased expression of the activation marker, CD83, as well as phosphorylation levels of AKT, PKC, ERK, p38, CAMKII and NFκB.^106^ Therefore, calcium plays an important role in mediating activation of B cells and future studies on the role of other calcium permeable channels in B‐cell activation are warranted.

### 5.2 Mg^2+^permeable ion channels

TRPM7 is a non‐selective cation channel that has been implicated in calcium influx and store recovery upon BCR stimulation,^70^ as well as Mg^2+^ homeostasis.^48^ We recently identified a key role for both the channel and kinase functions of TRPM7 in BCR signaling and activation.^107^ We found that both TRPM7‐KO and kinase‐dead (KD) DT40 B cells have increased and sustained ERK phosphorylation upon BCR stimulation. Similarly, primary murine B cells expressing only a single allele of *TRPM7* (*TRPM7^±^*) also exhibit increased and prolonged ERK phosphorylation upon BCR stimulation. Interestingly, addition of supplemental Mg^2+^ to TRPM7‐KO and KD DT40 B cells, which has been shown to help TRPM7‐KO cells overcome growth defects,^48^ restores the level of ERK phosphorylation in response to BCR stimulation to WT control cells. In addition, TRPM7‐KO DT40 B cells have a profound defect in internalizing the BCR upon stimulation, consistent with a role for TRPM7 in the internalization of other cell surface proteins including Fas receptor^108^ and Toll‐like receptor 4.^51^ In contrast, *TRPM7^±^* primary murine B cells internalize BCR comparable to WT cells, demonstrating that the channel, even at lower expression levels, is sufficient to restore downstream processes in B‐cell activation. Although BCR signaling was defective in cells expressing kinase dead TRPM7, these cells also internalized BCR comparable to WT cells, indicating that that kinase function of TRPM7 is not required for this specific process. We hypothesized that the requirement for the channel domain of TRPM7, but not the kinase function in BCR endocytosis may be linked to altered levels of PIP_2_, which are associated with impaired actin reorganization necessary for endocytosis,^109^ as we found TRPM7‐KO B cells had increased abundance of PIP_2_, but TRPM7‐KD cells did not. Interestingly, we found that supplementation with extracellular Mg^2+^ restored BCR signaling in TRPM7‐KO cells to WT levels, but was unable to rescue the defect in BCR internalization. The reason for this difference in the ability of supplementary Mg^2+^ to rescue these processes is not clear, but may be linked to the requirement of divalent cations for activity of DAG kinase ξ, which inhibits DAG signaling by phosphorylating DAG to produce phosphatidic acid and has been shown to regulate ERK signaling in mature FO B cells.^110^ It should also be noted that although supplementary Mg^2+^ was not sufficient to rescue the BCR internalization defect in TRPM7‐KO cells this finding does not rule out a possible role for Mg^2+^ in BCR endocytosis, as we cannot be sure that in the absence of TRPM7, uptake of Mg^2+^ by other magnesium permeable channels is sufficient to restore physiological levels in intracellular Mg^2+^. Importantly, however, we found that this defect in BCR‐antigen internalization was recapitulated in a B cell line treated with the TRPM7‐specific inhibitor, NS8593, and that this impacted on antigen presentation, as these B cells failed to activate T cells and induce IL‐2 production. This demonstrates the importance of magnesium homeostasis and potentially free cytoplasmic levels of magnesium in B cells upon stimulation.

Studies on a murine model deficient in another magnesium channel expressed in B cells, MAGT1, have also demonstrated defects in B‐cell activation.^85^ MAGT1‐deficient B cells exhibited reduced Mg^2+^ levels in both the steady‐state and upon BCR stimulation, but conversely increased Ca^2+^ flux. BCR stimulation of MAGT1‐KO B cells led to increased phosphorylation of Spleen tyrosine kinase (Syk), PLCγ2, and Protein kinase C beta II (PKCβII), and shortened period of phospho‐PKCδ signaling. In fact, BCR stimulation in the presence of IL‐4 enhanced proliferation of MAGT1‐deficient B cells, suggesting that albeit lower magnesium levels upon B‐cell activation, the enhanced calcium response observed leads to enhanced B‐cell activation. Thus, Mg^2+^ is emerging as an important regulator of BCR signaling and activation and both TRPM7 and MAGT1 appear to play important roles in regulating Mg^2+^ homeostasis in B cells.

### 5.3 Lysosomal ion channels

TRPML proteins are lysosomal calcium channels that are known to cause lysosomal storage defects when dysfunctional.^111^, ^112^ DT40 B cells express TRPML1 and 2, and although TRPML1‐KO DT40 B cells display no abnormalities in lysosomal structure, it has been speculated that TRPML2 can compensate for the lack of TRPML1.^113^ In contrast, TRPML2‐KO DT40 B‐cell generation was not viable, suggesting a potential role for this channel in B‐cell lysosome homeostasis and cell viability. Tagging either of these proteins with a C‐terminal GFP renders them nonfunctional, as demonstrated by identical lysosomal defects that arise from expression of these GFP‐tagged channel variants, further demonstrating functional redundancy. Stimulating TRPML1‐GFP and TRPML2‐GFP expressing DT40 B cells by BCR crosslinking led to BCR internalization where the endocytosed BCR complexes colocalize in the abnormal lysosomal structures. This finding highlights the need for normal lysosomal calcium homeostasis, as BCR‐Ag breakdown and loading onto MHC class II rely on healthy, functioning lysosomes within the B cell.

### 5.4 Zn^2+^ transporters

Zinc is not only crucial for immune cell development and specifically B‐cell development, but also humoral immunity. Indeed, zinc deficiency leads to immunodeficiency, suggesting impaired B‐cell activation and thus, a lack of antibody production. In addition, several gene deletion studies of Zn^2+^ transporters have identified an important role for these channels in B‐cell activation. For example, deficiency of ZIP9, a transporter of zinc from the Golgi to the cytosol, in DT40 B cells leads to reduced Akt and ERK phosphorylation upon BCR stimulation.^86^ In contrast, a DT40‐TKO of ZnT5/6/7, which transport Zn^2+^ from the cytosol to the Golgi, did not show this same signaling defect. Decreased ERK and Akt phosphorylation were found to be due, in part, to enhanced phosphatase activity in ZIP9‐KO B cells. Similarly, in mice expressing a ZIP7 (shuttles Zn^2+^ from ER to cytoplasm) point mutant that renders the transporter non‐functional, when crossed with mice expressing a transgenic hen egg lysozyme specific‐BCR (SW_HEL_) to bypass the developmental defect observed in ZIP7‐KO, BCR signaling is also impaired.^39^ Consistent with the ZIP9‐KO,^86^ decreased phosphorylation status of Syk, PLCγ2, and ERK upon BCR stimulation was due to decreased cytoplasmic zinc and consequently increased phosphatase activity in the ZIP7 mutant.^39^

ZIP10 is a plasma membrane localized transporter than can flux zinc ions into the cytoplasm of cells.^40^, ^42^ A conditional knockout of this transporter in APCs allowed for the study of ZIP10 and its role in B‐cell activation.^42^ ZIP10‐deficient B cells do not have lower resting state zinc levels, however zinc uptake is greatly impaired. This lack of uptake of extracellular zinc upon BCR stimulation led to hyperactivation of B cells, as Lyn, Syk, ERK, Akt, and NFκB phosphorylation was greatly enhanced. Notably, in contrast to ZIP7 and ZIP9, it was found that ZIP10 positively regulates phosphatase activity; CD45 activity was significantly reduced in ZIP10‐deficient B cells and supplementation with high levels of extracellular Zn^2+^ restored Lyn phosphorylation to levels comparable to the control cells. Interestingly, ZIP10‐deficient B cells fail to proliferate, possibly due to induction of apoptotic or anergic pathways due to hyperresponsiveness to stimuli.

Taken together, these studies demonstrate the important role of zinc in regulating B‐cell responses, primarily, it appears, through regulating phosphatase activity and thus BCR signaling. Further studies examining other zinc transporters expressed on B cells may help further delineate these roles and how dysregulation of zinc leads to immunodeficiency.

### 5.5 Monovalent cations

Monovalent cations are also very important to B‐cell activation, as the cell undergoes membrane potential changes rapidly upon BCR stimulation and efflux of these ions regulates membrane polarization and ROS production.^88^, ^89^, ^90^, ^91^ One group found that resting B cells preferably express voltage‐gated potassium channels and there is a switch in expression to calcium‐dependent channels in activated B cells.^93^ However, it has also been shown that BCR stimulation increases the expression of both types of channels, whereas LPS only induces voltage‐gated potassium channel expression.^94^ Blocking both K_Ca_3.1 and K_v_1.3 in B cells decreased ERK activation and severely decreases their potential to proliferate upon BCR stimulation.^114^ These findings may be correlated to reduced Ca^2+^ influx due to lack of K^+^ efflux and loss of the driving force needed for calcium to enter the cells.

Proton currents that are voltage dependent have been recorded in B cells^115^ and knocking out *HVCN1* demonstrated how important these currents are during B‐cell activation.^91^ This protein is found in the BCR cap upon stimulation and is internalized with the BCR. H_V_1 deficiency led to diminished ROS production and reduced SHP‐1 oxidation, which is crucial to inhibiting its negative regulatory effect during B‐cell activation. Due to reduced SHP‐1 oxidation, global tyrosine phosphorylation of proteins was decreased in H_V_1‐deficient B cells. Specifically, Syk and Akt phosphorylation were significantly reduced, however ERK and calcium signaling were comparable to WT cells if not slightly increased. In addition, H_V_1‐deficient cells produced less energy, had reduced metabolism and did not proliferate as well as WT B cells. This demonstrates the crucial role for proton efflux upon BCR stimulation, as reduced ROS production leads to impaired activation. In contrast, some B cell lines express a truncated H_V_1 protein that is 20 amino acids shorter and is much more active.^116^ Overexpressing this protein form in murine B cell lines resulted in increased ERK activation and surprisingly, increased proliferation upon BCR stimulation. Additionally, overexpressing full‐length H_V_1 in A20 murine B cells impaired antigen presentation due to reduced acidification of lysosomes, as this protein is endocytosed along with BCR‐Ag complexes and causes proton efflux into the cytosol from the lysosome.^91^ These findings demonstrate the importance of proton currents both at the plasma membrane and in MHC‐class II positive lysosomes in B‐cell activation. Loss of these currents, as well as enhanced currents both dysregulate B‐cell activation and can have major impacts on humoral immunity.

## 6 ION CHANNELS IN EARLY B‐CELL ACTIVATION AND SYNAPSE FORMATION

The first step in B‐cell activation requires B cells to find and bind to antigen through the BCR. This is facilitated by the trafficking of B cells through secondary lymphoid organs such as the spleen and lymph nodes, scanning cells for the presence of antigen. B cells predominantly recognize antigen in membrane‐bound form present on the surface of APCs including macrophages and dendritic cells.^117^ Upon BCR recognition of membrane‐bound antigen the B cell spreads rapidly over the antigen‐bearing membrane to try and bind to as much antigen as possible, forming multiple discrete “microclusters” of BCR and antigen before contracting and gathering the antigen into a central cluster.^118^ BCR‐Ag microclusters that are formed are sites of signaling where tyrosine kinases act on their substrates to potentiate the signaling cascade.^119^, ^120^ Importantly, these early events not only dictate the propensity of the B cell being activated, but also the degree of antigen that is gathered, internalized, and presented to T cells and thus, the degree of T cell help the B cell receives.^118^ In this section, we discuss the role of ions and ion channels important in these early processes of B‐cell activation.

### 6.1 Calcium channels

Calcium is known to be an important secondary messenger for motility of cells, including lymphocytes.^121^, ^122^ In the context of B cells, a sodium‐calcium exchanger, NCLX, on mitochondrial membranes was found to play an important role to B‐cell migration.^123^ This channel effluxes Ca^2+^ from stores located in the mitochondria in exchange for Na^+^ in the cell. RNA silencing of this channel in both A20 murine B cells and chicken DT40 B cells increased random cell movement and migration which was not altered by addition of the chemokine, CXCL12. Interestingly, NCLX‐silenced B cells had higher basal levels of cytosolic calcium but reduced Ca^2+^ flux upon addition of CXCL12 in comparison to control cells. In addition, NCLX knockdown altered F‐actin organization and Rac1 localization, consistent with lack of directional migration, which appeared to depend on mitochondrial polarization. Inhibition of NCLX in primary murine B cells recapitulated chemotaxis defects and reduced calcium response upon BCR stimulation due to ER calcium leak into the cytosol. Therefore, regulation of cytosolic Ca^2+^ is crucial for normal cell motility and chemotaxis, which is critical for B cells to traffic to secondary lymphoid organs surveying for cognate antigen.

It has also been found that ion channels mediate inside‐out integrin activation, which is crucial for B cells to traffic to lymphoid organs,^124^ as well as mediate B‐cell interactions with APCs.^117^, ^125^ Ligands that activate non‐store‐operated non‐selective cation channels on B cells allowed for B‐cell binding to VCAM‐1 and ICAM‐1.^124^ This was found to be dependent on their ability to regulate the membrane potential and not due to their ability to influx calcium. During mechanical stress, it was found that both NSCCs and SOCE currents were activated, suggesting an overlapping signaling pathway which involves PLCγ2. However, BCR stimulation attenuates NSCC currents, suggesting that currents from NSCCs such as TRPV4 are active during steady‐state and may help B cells migrate, whereas integrin activation upon BCR stimulation to mediate attachment to APCs may rely on CRAC channel currents. These findings demonstrate a key role of calcium and ion channel currents in inside‐out integrin activation in response to varied stimuli.

Calcium has also been found to be important in B‐cell actin reorganization and B‐cell spreading upon recognition of antigen. For example, inhibition of CRAC channels with IP_3_R antagonist 2‐APB (2‐aminoethoxydiphenyl borate) prevented A20 and primary murine B cells from adhering to anti‐Ig‐coated coverslips.^126^ These cells do not produce radial lamellipodia and fail to spread in response to stimulation. Calcium ionophore treatment of these cells induces higher cytosolic calcium levels and remodeling of the actin cytoskeleton. Inhibition of calcium influx with 2‐APB, on the other hand, prevented dephosphorylation of the actin disassembling protein cofilin, which is necessary for B‐cell spreading and microcluster formation.^127^ Therefore, calcium influx by ion channels is crucial to the B‐cell spreading response.

### 6.2 TRP channels

We recently identified an important role for TRPM7 in regulating the actin cytoskeleton and consequently several early events in B‐cell activation.^107^ We demonstrated that deletion of *TRPM7* in DT40 B cells led to cytoskeletal defects both at resting state and upon activation, as TRPM7‐KO B cells exhibited elongated filopodia‐like protrusions, but reduced lamellipodia when cells are adhered to glass. However, in a planar lipid bilayer system with anti‐Ig as surrogate antigen, TRPM7‐KO B cells spread over a larger area and form an increased number of BCR‐antigen microclusters compared to WT cells, but failed to centrally aggregate the antigen as efficiently as WT B cells. This finding was recapitulated in primary murine B cells expressing only one allele of *TRPM7*. Importantly, supplementation with high concentrations of extracellular Mg^2+^ was not able to rescue the defect in B‐cell contraction and centralization of antigen. We also investigated the role of the kinase function of TRPM7 and found that DT40 B cells expressing kinase dead TRPM7 (TRPM7‐KD) also exhibited cytoskeletal defects, and also failed to centralize antigen. Interestingly, however, these cells differed from TRPM7‐KO cells as they were slower to spreading and had even higher antigen accumulation. The centralization of antigen was also more severely compromised in TRPM7‐KD cells as BCR‐antigen microclusters were largely immobilized where they formed and did not come together in one large aggregate as in TRPM7‐KO B cells. Differences in microcluster aggregation may be in part due to the failure of actin clearance from the center of the contact with the antigen‐containing bilayer in TRPM7‐KD cells, whereas actin clearance did not appear to be affect in TRPM7‐KO cells. Surprisingly, in contrast to TRPM7‐KO cells, the defect in the centralization of antigen did not lead to increased total antigen intensity at the contact, suggesting internalization of antigen is not impaired when the channel is still active. We hypothesized that the phenotypic differences we observed between TRPM7‐KO and TRPM7‐KD cells may be due to channel functionality being intact in KD cells, but reduced phosphorylation of PLCγ2 as it is a target of TRPM7 kinase.^73^ Therefore, magnesium seems to play a key role in antigen internalization, whereas the kinase domain is required for proper spreading and contraction.

## 7 ION CHANNELS IN B‐CELL EFFECTOR FUNCTIONS

**Figure 4:**
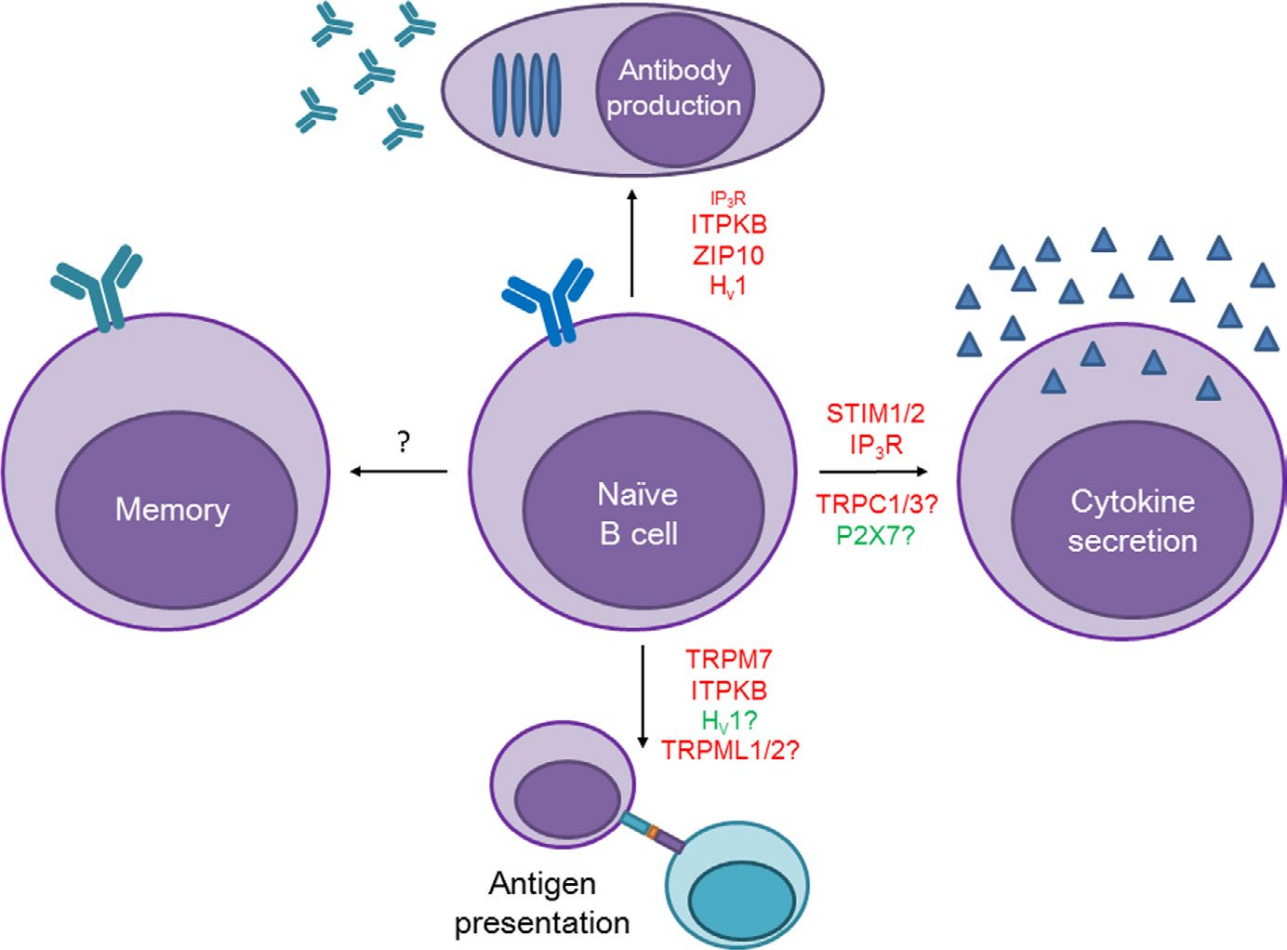
Ion channels regulate a multitude of B‐cell functions. Naive circulating B cells can be activated by recognition and binding of specific antigen to their B‐cell receptor (BCR). These B cells internalize this antigen and present it to provide T cell help (bottom). Knockout models of TRPM7 and ITPKB demonstrated impaired antigen presentation through reduced surface expression of costimulatory molecules and MHC class II. The overexpression of H_v_1 was found to impair antigen presentation, as this channel is internalized with BCR‐Ag complexes and prevents lysosomes from acidifying. TRPML1/2 mutants may impact antigen presentation due to associated defects in lysosomal. Activated B cells can differentiate into plasma cells (top), which secrete antibodies to tag pathogen for removal. IP_3_R knockout mice have impaired GC B‐cell numbers and antibody production in the first week postimmunization, but recovered to normal levels by week 2. ITPKB knockout mice had altered antibody responses, as basal antibody levels were increased, but responses postimmunization were severely impaired. ZIP10 knockout mice also have impaired antibody responses, which mirror defects found in mice on zinc‐deficient diets. H_V_1 knockout mice also have impaired antibody responses and defective class switching. B cells can also regulate immune responses through secreting various cytokines (right). Dysregulated calcium influx in STIM1/2 and IP_3_R knockout B cells upon BCR stimulation led to decreased NFAT activity and thus, lower IL‐10 secretion. TRPC1‐ and TRPC3‐deficient B cells also have decreased NFAT activity, suggestive of decreased IL‐10 secretion. P2X7 dampens calcium responses through sodium influx upon BCR stimulation in the presence of ATP and loss of this channel may lead to enhanced IL‐10 secretion due to enhanced calcium and NFAT activity. B cells can differentiate into memory B cells after activation (left). Currently, it is unknown whether any ion channels play a role in memory B‐cell generation and further research is needed to understand their role in regulating this process. Key regulators that inhibit B‐cell functions are highlighted in red or enhance B‐cell functions in green. The degree of impact on B‐cell functions are indicated by font size, with more severe impact in larger font and more modest impact represented by a smaller font

B‐cell activation leads to differentiation into plasma cells that secrete antibodies and provide lasting humoral immunity, or memory cells that can be activated quicker upon re‐exposure to the same antigen. B cells also produce and secrete cytokines that can govern local immune responses. Ion dysregulation can lead to improper antibody responses in vivo and defective humoral immunity, as well as impaired cytokine secretion that can impact immune cell responses to various pathogens (Figure 4).

### 7.1 Calcium channels

Calcium plays a key role in B‐cell secretion of IL‐10, an anti‐inflammatory cytokine, as both the DKO of STIM1/2 and TKO of IP_3_Rs in mice led to defective IL‐10 production after B‐cell activation.^60^, ^65^ Regulatory B‐cell numbers and IL‐10 secretion were reduced in IP_3_R‐TKO mice, which was attributed to poor induction of the calcineurin‐NFAT pathway upon BCR stimulation.^65^ In contrast, regulatory B‐cell numbers were not impacted by STIM1/2 deficiency; however, IL‐10 mRNA transcription was downregulated and IL‐10 secretion by this B‐cell subset was impaired as seen in IP_3_R‐TKO cells.^60^ Therefore, lack of calcium influx by B cells upon activation can impair their ability to negatively regulate immune responses in vivo.

Surprisingly, antibody production does not have the same dependence on calcium influx during B‐cell activation as cytokine secretion does. IP_3_R‐TKO mice had normal levels of IgM, IgG, and IgA serum titers in unimmunized mice; however, immunization with the T‐dependent antigen, NP‐CGG, led to reduced germinal center (GC) B‐cell numbers, as well as lower IgM and IgG1 antibody titers.^65^ However, by week 2 postimmunization, these mice had comparable levels of serum antibody titers as WT controls. IgM responses upon immunization with a T‐independent antigen exhibited a similar trend, as week 1 titers were slightly less than WT, but recovered by week 2. Interestingly, IgM antibody titers in response to LPS were slightly higher than WT controls, but also dropped to being comparable to WT controls by week 2.

Consistent with IP_3_R‐TKO mice, STIM1/2‐DKO mice also have normal levels of basal Igs.^60^ However, these mice had comparable numbers of GC B cells and affinity maturation as WT B cells when challenged with a T‐dependent antigen. This was reflected in lack of a difference in IgM and IgG1 antibody responses over 7 weeks even after a boost immunization. Similarly, responses to T‐independent antigens were comparable across all timepoints for IgM and IgG3 in STIM1/2‐DKO and WT mice. These findings demonstrate that the sustained phase of calcium is dispensable for antibody production; however, IP_3_R‐KO B cells did have reduced GC B cells and slight differences in antibody production in the first week post immunization,^65^ demonstrating that even minor differences in calcium signaling can lead to varying antibody production in initial stages.

In contrast to reduced calcium signaling, enhanced calcium signaling had strong impacts on antibody production in vivo. ITPKB‐KO mice had significantly elevated basal serum titers of IgM, markedly reduced titers of IgG1 and IgA and no difference in IgG3 titers in comparison to WT mice.^66^ These mice do have higher numbers of B1 B cells and lower numbers of T cells, which may reflect the differences in basal antibody titers. Interestingly, there was no difference in IgM titers 1 week postimmunization with a T‐independent antigen despite higher basal levels; however, there was a substantial reduction in IgG3 after 1 week. This demonstrates the crucial role of fine‐tuning calcium responses during B‐cell activation, as hyperresponsiveness and increased calcium leads to dysregulated antibody production in some contexts.

### 7.2 Unconventional ions

Magnesium homeostasis is essential for B‐cell development, maintenance, and activation. Conditional knockout of TRPM7 in B cells results in a developmental block at the pro‐B‐cell stage,^45^ which has made it difficult to study the importance of magnesium in humoral immune responses. It has been shown, however, that MAGT1‐KO mice have impaired plasma cell numbers preimmunization.^128^ MAGT1 was found to partially rescue B‐cell development phenotypes in DT40 B cells^53^; however B‐cell development is largely unaffected in MAGT1‐deficient mice,^128^ whereas loss of TRPM7 completely abrogates B‐cell development from the pre‐B‐cell stage.^45^ These findings suggest that even minor dysregulation of magnesium homeostasis in B cells could impact their function.

Zinc has also been found to be very important in humoral immunity, as dysregulated homeostasis of this divalent cation leads to immunodeficiency. Patients with ZIP7 mutations and mouse models with a knock‐in mutation of ZIP7 both have very low or non‐detectable levels of basal serum titers of IgG and IgM, respectively.^39^ Mice with knock‐in ZIP7 mutations never survived long enough to determine basal serum IgG levels. A B‐cell conditional knockout of ZIP10 also demonstrated that dysregulated zinc homeostasis effects humoral immunity.^42^ Although there was no change in GC B cells in ZIP10‐cKO mice upon challenge with a T‐dependent antigen, IgM and IgG1 antibody responses were severely impaired. Surprisingly, B cells were still capable of class switch recombination when tested in vitro. Immunization with T‐independent antigens also resulted in defective antibody responses as IgM and IgG3 production were severely impaired in ZIP10‐KO mice. These findings were mirrored in WT mice fed a diet deficient in zinc, highlighting the key role of zinc ions for humoral immunity. Thus, dysregulation of zinc in B cells leads to a severe immunocompromised phenotype.

Generation of reactive oxygen species (ROS) is important for inhibiting phosphatase activity during B‐cell activation.^91^ It is also important for promoting B‐cell proliferation and downstream activation, as impairment of ROS production leads to less upregulation of activation markers on the cell surface. Proton efflux upon BCR stimulation has been found to be a contributing factor to ROS generation in B cells, as knocking out *HVCN1* reduced ROS production and reduced B‐cell activation. Even though GC B cells downregulate H_V_1, lack of this channel during the early events of B‐cell activation impacted antibody responses. Mice deficient in H_V_1 had reduced antibody titers of IgG1, IgG2b, and IgG3 upon immunization with a T‐dependent antigen; however, IgM antibody titers were comparable to WT, suggesting lack of H_V_1 impairs isotype switching. Interestingly, staining for antigen‐specific plasma cells and GCs demonstrated that GC reactions proceeded normally in H_V_1 deficient mice, which was supported by evidence of unaffected affinity maturation and normal GC sizes and cell numbers. Therefore, defects in isotype switching were due to plasma cell and plasmablast intrinsic defects. Immunization with a T‐independent antigen also led to defective antibody production in *HVCN1*‐KO cells, as titers of both IgM and IgG3 were reduced over a 2‐week period in comparison to WT cells. This demonstrates the requirement for ROS production in B cells and the inhibition of phosphatases to allow for robust B‐cell activation and antibody production in vivo.

Potassium channel expression has been observed to increase upon B‐cell activation.^93^, ^94^, ^95^ These channels are active in naive B cells, suggesting upregulation of channel expression is needed for enhanced potassium currents in activated cells. Memory B cells are thought to contribute to pathogenesis of autoimmune disorders and potassium channel activity in these cells may potentiate this.^95^ Memory B cells with class‐switched BCRs (IgD^−^) have enhanced potassium efflux currents from both voltage‐gated (K_v_1.3) and calcium‐activated (K_Ca_3.1) potassium channels in comparison to naive, and IgD^−^CD27^−^ and IgD^+^CD27^+^ memory B cells. IgD^−^CD27^−^ memory B cells had no change in potassium channel expression, whereas IgD^+^CD27^+^ memory cells express more K_Ca_3.1 channels compared to naive B cells, but similar levels of K_v_1.3. IgD^−^CD27^+^ memory B cells have higher density (accounts for larger size) of both potassium channels. Interestingly, activation of IgD^−^CD27^−^ and IgD^+^CD27^+^ memory B cells led to increases in channel density of K_Ca_3.1 but no change in K_v_1.3, whereas activation induced no change in potassium channel density in IgD^‐^CD27^+^ memory B cells. Blocking K_Ca_3.1 activity in IgD^+^CD27^+^ memory B cells prevented proliferation, while blocking K_v_1.3 activity prevented proliferation of IgD^−^CD27^+ ^memory B cells. This demonstrates the significance of potassium ion efflux upon re‐activation of different populations of memory B cells and that dysregulation of these channels leads to abrogated proliferation, which may impact immune responses toward secondary infections.

## 8 ION CHANNELS IN HEALTH AND DISEASE

Much of the progress in our understanding of ion channels stems from observations of human patients with ion deficiencies, either due to nutritional deficiencies or genetic mutations in ion channels. Mutations in genes encoding ion channel components result in a wide range of “channelopathies,” diseases that affect a variety of physiological processes and range from mild to life‐threatening.^129^ In the context of the immune response, the discovery of patients with severe combined immunodeficiencies due to mutations in CRAC channel components^63^, ^64^, ^130^, ^131^ and *MAGT1* 128 has facilitated and spurred the study of ion channels and ion signaling in the immune system. The study of these patients, as well as the generation of murine models with targeted deletion of genes encoding ion channels and transporters have helped identify many key roles of ions, the critical ion channels/transporters regulating these ions, and their important function in the immune response.

Although the primary immunodeficiencies associated with mutations in ion channel genes so far identified are largely associated with defects in T cells, the evidence gathered from murine models discussed here clearly demonstrate an important role for ion channels/transporters in B‐cell biology. Notably, some of the first evidence for a role for ions in B‐cell biology came from observations of patients not with genetic mutations in ion channels, but rather ion deficiencies due to malnutrition,^132^ which is surprisingly common throughout the world. Indeed, it is estimated that 20%‐30% of the global population are zinc deficient,^133^ but for some subregions the estimates are >70%. Similarly, up to 75% are magnesium deficient.^134^ These deficiencies resulting from malnutrition are rampant not only in developing countries, but also surprisingly common in developed countries; an estimated 50% of the US population do not meet the recommended daily allowance of magnesium.^135^ In addition to dietary deficiencies, patients with malabsorption issues caused by intestinal or colon damage, such as Crohn's, irritable bowel syndrome, and celiac disease are frequently deficient in critical ions.^136^ Moreover, many of the therapies used to treat a variety of diseases lead to ion deficiencies; for example, digoxin for the treatment of heart conditions, or the chemotherapeutic cisplatin, can lead to Mg^2+^ deficiencies.^137^, ^138^, ^139^ Thus, continued effort to elucidate the role of ion signaling, and the characterization of varied ion channels in immune cells, is fundamental in improving our understanding of human health and disease, and possibly the potential to improve human disease conditions through targeting ion channels with pharmacological inhibitors (for which several have already been identified) or nutritional ion deficiencies.

## 9 FUTURE PERSPECTIVES

The study of ion channels and ion signaling in lymphocytes, and particularly in B cells, is in its infancy. Granted, we have learned much about Ca^2+^ as a second messenger and the discovery of STIM and ORAI proteins was a key to elucidating how Ca^2+^ fluxes are regulated. Yet, these proteins are found to be almost dispensable in B‐cell development and activation. This suggests that other ion channels in B cells contribute to calcium regulation during these stages that have yet to be explored. In addition, new research into other ions, such as Mg^2+^ and Zn^2+^, have led to surprising new discoveries of the critical role of these ions in B‐cell development, activation, differentiation, and effector function, and the ion channels important in the regulation of these ions are being identified. However, B cells express a wealth of ion channels which remain completely unexplored in the context of B‐cell biology. In addition, our knowledge of how these different ion channels work in concert to fine‐tune B‐cells responses is not well understood. We are also just beginning to unravel the impact of genetic mutations/SNPs and protein expression changes of ion channels in B cells and how these can contribute to disease. Understanding the complex network of ion signaling during the varying stages of B cell “life” could help in the development of therapeutics to treat B‐cell diseases.

## CONFLICT OF INTEREST

The authors have no conflicting financial interests.
